# Supplementary material for: Using a data-driven approach for the development and evaluation of phenotype algorithms for systemic lupus erythematosus
Source: PLoS One. 2023 Feb 16;18(2):e0281929. doi: 10.1371/journal.pone.0281929 (PMC9934349; doi:10.1371/journal.pone.0281929)
Supplement: S1 File — (DOCX) [file pone.0281929.s001.docx]

Supplemental Information 1: Literature search and results

1. Search criteria:

Our search strategy included the MeSH term systemic lupus erythematosus , and the terms “retrospective cohort” epidemiology [MeSh Term], “epidemiologic Methods” [MeSH Term], phenotype, “validation study”, “positive predictive value”, “sensitivity and specificity”, “insurance database”, “claims database”, “administrative database”, and “algorithm”. We also included the terms for many of the databases that are used in validation studies and other key words: Medicaid, Medicare, Truven, Optum, Medstat, Marketscan, “Nationwide Inpatient Sample”, “National Inpatient Sample”, PharMetrics, PHARMO, ICD-9, ,ICD-10, “electronic medical records” , “Veterans Affairs, “ Premier database”, “National Health Insurance Research Database”, "administrative claims", "General Practice Research Database", “Clinical Practice Research Datalink” and “Health Improvement Network”.

1. Retrieved articles:

| Author | Citation |
| --- | --- |
| Klein | Vaccine. 2010 Jan 22;28(4):1062-8. |
| Karve | J Pediatr. 2012 Oct;161(4):662-670.e2. |
| Hermansen | J Rheumatol. 2016 Jul;43(7):1335-9. |
| Tanaka | Int J Rheum Dis. 2018 Aug;21(8):1609-1618. |
| Yun | PLoS One. 2017 Jun 27;12(6):e0179088. |
| de Abreu | Semin Arthritis Rheum. 2018 Apr;47(5):749-755. |
| Hesselvig | Lupus. 2017 Jan;26(1):48-53. |
| Harris | Lupus. 2016 Aug;25(9):1045-9 |
| Gandelman | Arthritis Care Res (Hoboken). 2019 Mar 15. |
| Turner | BMC Med Inform Decis Mak. 2017; 17: 126. |
| Barnado | Arthritis Care Res (Hoboken). 2017 May;69(5):687-693. |
| Barnado | Lupus. 2019 Jan;28(1):66-76. |
| Son | Pediatrics. 2014 Jan;133(1):e106-13. |
| Bernatsky | J Rheumatol. 2011 Aug;38(8):1612-6. |
| Chibnik | Lupus. 2010 May;19(6):741-3. |
| Barbhaiya | Semin Arthritis Rheum. 2019 Apr;48(5):840-846. |
| Barbhaiya | Arthritis Rheumatol. 2017 Sep;69(9):1823-1831. |
| Chen | BMJ Open. 2019 Jun 19;9(6):e027495. |
| Chen | Arthritis Care Res (Hoboken). 2015 Aug;67(8):1086-94. |
| Chiu | Rheumatol Int. 2016 Nov;36(11):1507-1514. |
| Feldman | Semin Arthritis Rheum. 2018 Oct;48(2):205-213. |
| Gomez-Puerta | Arthritis Rheumatol. 2015 Mar;67(3):752-60. |
| Feldman | Arthritis Rheumatol. 2015 Jun;67(6):1577-85. |
| Goss | PLoS One. 2015 Jun 18;10(6):e0128920. |
| Feldman | Arthritis Care Res (Hoboken). 2015 Dec;67(12):1712-21. |
| Feldman | Arthritis Rheum. 2013 Mar;65(3):753-63. |
| Hiraki | Arthritis Care Res (Hoboken). 2017 Nov;69(11):1620-1626. |
| Knight | J Rheumatol. 2014 Mar;41(3):539-46. |
| Herrinton | J Rheumatol. 2016 Aug;43(8):1503-9. |
| Knight | J Rheumatol. 2016 Jul;43(7):1427-33. |
| Chang | Lupus. 2018 Nov;27(13):2146-2154. |
| Hiraki | Arthritis Rheum. 2012 Aug;64(8):2669-76. |
| Li | Arthritis Rheum. 2009 Jun 15;61(6):755-63. |
| Murray | PLoS One. 2016 Jan 5;11(1):e0144918. |
| Petri | J Med Econ. 2015;18(11):967-73. |
| Ling | Pediatr Rheumatol Online J. 2018 Apr 16;16(1):26. |
| Lin | Arthritis Care Res (Hoboken). 2018 Nov;70(11):1700-1706. |
| Tanzer | Arthritis Care Res (Hoboken). 2013 Mar;65(3):382-90. |
| Tektonidou | Arthritis Care Res (Hoboken). 2015 Aug;67(8):1078-85. |
| Tektonidou | Arthritis Rheumatol. 2016 Nov;68(11):2680-2685. |
| Scalzi | Arthritis Rheum. 2010 Sep;62(9):2767-75. |
| Yang | J Rheumatol. 2011 Aug;38(8):1612-6. |
| Yazdany | Arthritis Rheumatol. 2014 Oct;66(10):2828-36. |
| Walunas | Arthritis Care Res (Hoboken). 2017 Sep;69(9):1369-1376. |
| Jorge | Semin Arthritis Rheum. 2019 Aug;49(1):84-90. |
| Garris | J Med Econ. 2013;16(5):667-77. |
| Kabadi | Lupus. 2018 Oct;27(11):1799-1809. |
| Khaliq | Breast Cancer Res Treat. 2015 Jun;151(2):465-74. |
| Furst | Lupus 2013 22: 99 |
| Kan | Clin Ther. 2016 Mar;38(3):610-24. |
| Kan | Biomed Res Int. 2013;2013:808391. |
| Oglesby | Appl Health Econ Health Policy. 2014 Apr;12(2):179-90. |
| Feldman | Lupus. 2017 Jun;26(7):682-689. |
| Hanly | Lupus. 2014 Nov;23(13):1377-82 |
| Rees | Ann Rheum Dis. 2016 Jan; 75(1): 136–141. |
| Moores | Vaccine. 2013 Dec 30;31 Suppl 10:K62-73. |
| Arkema | BMJ Open. 2016 Jan 4;6(1):e007769. |
| Nielsen | Hum Reprod. 2011 Jun;26(6):1555-9. |

1. Resultant diagnosis codes from articles found in search:

| Article # | code | Vocabulary |
| --- | --- | --- |
| 1 | N000.00 | Read Codes |
| 1 | N000000 | Read Codes |
| 1 | N000100 | Read Codes |
| 1 | 695.4 | ICD-9 |
| 1 | 710.0 | ICD-9 |
| 1 | 373.34 | ICD-9 |
| 2 | 695.4 | ICD-9 |
| 2 | 710 | ICD-9 |
| 3 | DM321 | ICD-10 |
| 3 | DM328 | ICD-10 |
| 3 | DM329 | ICD-10 |
| 4 | M32 | ICD-10 |
| 5 | M32 | ICD-10 |
| 6 | M32 | ICD-10 |
| 6 | L93 | ICD-10 |
| 7 | M32 | ICD-10 |
| 8 | 734.1 I | ICD-8 |
| 8 | 710 | CD-9 |
| 8 | M32 | ICD-10 |
| 9 | 710.0 | ICD-9 |
| 10 | 710.0 | ICD-9 |
| 11 | 710.0 | ICD-9 |
| 12 | 710.0 | ICD-9 |
| 13 | 710.0 | ICD-9 |
| 14 | 710.0 | ICD-9 |
| 15 | 710.0 | ICD-9 |
| 16 | 710.0 | ICD-9 |
| 17 | 710.0 | ICD-9 |
| 18 | 710.0 | ICD-9 |
| 19 | 710.0 | ICD-9 |
| 20 | 710.0 | ICD-9 |
| 21 | 710.0 | ICD-9 |
| 22 | 710.0 | ICD-9 |
| 23 | 710.0 | ICD-9 |
| 24 | 710.0 | ICD-9 |
| 25 | 710.0 | ICD-9 |
| 26 | 710.0 | ICD-9 |
| 27 | 710.0 | ICD-9 |
| 28 | 710.0 | ICD-9 |
| 29 | 710.0 | ICD-9 |
| 30 | 710.0 | ICD-9 |
| 31 | 710.0 | ICD-9 |
| 32 | 710.0 | ICD-9 |
| 33 | 710.0 | ICD-9 |
| 34 | 710.0 | ICD-9 |
| 35 | 710.0 | ICD-9 |
| 36 | 710.0 | ICD-9 |
| 37 | 710.0 | ICD-9 |
| 38 | 710.0 | ICD-9 |
| 39 | 710.0 | ICD-9 |
| 40 | 710.0 | ICD-9 |
| 41 | 710.0 | ICD-9 |
| 42 | 710.0 | ICD-9 |
| 43 | 710.0 | ICD-9 |
| 44 | 710.0 | ICD-9 |
| 45 | 710.0 | ICD-9 |
| 45 | M32.1 | ICD-10 |
| 46 | 710.0x | ICD-9 |
| 47 | 710.0x | ICD-9 |
| 48 | 710.0x | ICD-9 |
| 49 | 710.0x | ICD-9 |
| 50 | 710.0x | ICD-9 |
| 51 | 710.0x | ICD-9 |
| 52 | 710.0x | ICD-9 |
| 53 | 710.x | ICD-9 |
| 54 | 710.0 | ICD-9 |
| 54 | M 32 | ICD-10 |
| 54 | M32.1 | ICD-10 |
| 54 | M32.8 | ICD-10 |
| 54 | M32.9 | ICD-10 |
| 55 | N000300 | Read Codes |
| 55 | N000400 | Read Codes |
| 55 | N000600 | Read Codes |
| 55 | N000z00 | Read Codes |
| 55 | Nyu4300 | Read Codes |
| 55 | M154.00 | Read Codes |
| 55 | M154000 | Read Codes |
| 55 | M154100 | Read Codes |
| 55 | M154200 | Read Codes |
| 55 | M154300 | Read Codes |
| 55 | M154400 | Read Codes |
| 55 | M154500 | Read Codes |
| 55 | M154600 | Read Codes |
| 55 | M154700 | Read Codes |
| 55 | M154z00 | Read Codes |
| 55 | Myu7800 | Read Codes |
| 55 | F371000 | Read Codes |
| 55 | F4D3300 | Read Codes |
| 55 | H57y400 | Read Codes |
| 55 | K01x400 | Read Codes |
| 55 | K01x411 | Read Codes |
