# Supplementary material for: Using a data-driven approach for the development and evaluation of phenotype algorithms for systemic lupus erythematosus
Source: PLoS One. 2023 Feb 16;18(2):e0281929. doi: 10.1371/journal.pone.0281929 (PMC9934349; doi:10.1371/journal.pone.0281929)
Supplement: S2 File — (DOCX) [file pone.0281929.s002.docx]

**Supplemental Information 2: Overview of Phenotype Algorithm Development Process**

Development of draft items

Selection of ischemic stroke as safety outcome

Scoping review for ischemic stroke

Refinement of items through Delphi process

Development of User Guide for tool

Stakeholder Assessment of tool

Operationalization of tool for further refinement

**Final ACE-IT Tool**

Scoping Review

*(Selection of Stroke Studies)*

Regulatory guidance documents, & other pre-existing literature

The algorithm development process begins with a literature search. The search algorithm used in PUBMED is detailed in Supplemental Information 1. We found 58 journal articles using phenotype algorithms for SLE. After reviewing the results from the literature search and determining all the diagnosis codes for SLE in the different vocabularies, i.e., International Classification of Diseases, Ninth (ICD-9) or Tenth (ICD-10) Revision and Read codes, codes were translated each into the Systemized Nomenclature of Medicine (SNOMED) vocabulary using the Observational Health Data Sciences and Informatics (OHDSI) open-source ATLAS tool (https://github.com/OHDSI/Atlas). Translating codes from disparate vocabularies into SNOMED has been shown to be effective and improves the efficiency and transportability of research.(1) We used the open-source PHOEBE tool (<https://data.ohdsi.org/PHOEBE/>) to search for possible missing terms using the data on code utilization in the OHDSI Network. We used the cohort diagnostic tool (https://github.com/OHDSI/CohortDiagnostics) to determine whether there were missing (“orphan”) codes based on keyword and synonym searching. After review of possible additional codes from PHOEBE and Cohort Diagnostics, we determined the final code set for the cohort definitions. We used the PheValuator tool (<https://github.com/OHDSI/PheValuator>) to determine the performance characteristics, i.e., sensitivity, specificity, and positive and negative predictive value, of the algorithms. The steps in the PheValuator process are described below. Finally, we again use the cohort diagnostic tool to determine cohort characteristics to evaluate whether index date misclassification may have occurred and whether algorithms demonstrate issues with specificity. This involves an iterative process where the algorithms are altered based on the findings. For example, if index date misclassification is determined to exist, an altered cohort where the index date is reclassified based on conditions present prior to the previous index indicated a possible earlier start to SLE.

Iterate on algorithms based on cohort diagnostics results

Determine algorithm performance characteristics with PheValuator (https://github.com/OHDSI/PheValuator)

Literature Review (details in Supplemental Information 1)

Convert native classification codes to SNOMED using ATLAS

(https://github.com/OHDSI/Atlas)

Orphan concept search using Cohort Diagnostics

(https://github.com/OHDSI/CohortDiagnostics)

Assessment of algorithms using Cohort Diagnostics

(https://data.ohdsi.org/SLECohortDiagnostics)

Development of algorithms

Final phenotype algorithms determined and assessed

PHOEBE Search

(https://data.ohdsi.org/PHOEBE/)

PheValuator process:

PheValuator (https://github.com/OHDSI/PheValuator) is a methodology within the OHDSI tool stack that allows researchers to determine the performance characteristics, i.e., sensitivity, specificity, and positive and negative predictive value, of phenotype algorithms. The method uses diagnostic predictive modeling to create a probabilistic reference standard to evaluate algorithm performance. The method involves a multi-step process. In the first step, a diagnostic predictive model is developed comparing cases and non-cases of the health condition using LASSO regularized regression and the Patient Level Prediction tool within the OHDSI tool stack (<https://github.com/OHDSI/PatientLevelPrediction>). The cases are determined using a very specific phenotype algorithm that extracts subjects with a high probability of being a case for the health condition. The non-cases are determined by extracting a random set of subjects for the entire dataset after removing any subjects with a moderate probability of being a case using a very sensitive phenotype algorithm for the health condition. The data to inform the model is from subject demographics, e.g., sex and age, subject health conditions, clinical observations, laboratory measurements, and drug exposures extracted from each subject’s record. The tool randomly selects a large set of subjects from the database, perhaps as many as two million subjects, and applies the regression model to this large group after extracting the same information as was extracted for model development. After applying the model, each the large set of subjects now has a determined probability of the health condition of interest. These probabilities are used to determine the performance of the algorithms. The full details of the process may be found at the link provided above.

1. Hripcsak G, Levine ME, Shang N, Ryan PB. Effect of vocabulary mapping for conditions on phenotype cohorts. J Am Med Inform Assoc. 2018;25(12):1618-25.
